# Rational design of multi epitope-based subunit vaccine by exploring MERS-COV proteome: Reverse vaccinology and molecular docking approach

**DOI:** 10.1371/journal.pone.0245072

**Published:** 2021-02-03

**Authors:** Usman Ali Ashfaq, Saman Saleem, Muhammad Shareef Masoud, Matloob Ahmad, Nazia Nahid, Rashid Bhatti, Ahmad Almatroudi, Mohsin Khurshid

**Affiliations:** 1 Department of Bioinformatics and Biotechnology, Government College University, Faisalabad, Pakistan; 2 Department of Chemistry, Government College University, Faisalabad, Pakistan; 3 Center of Excellence in Molecular Biology, University of the Punjab, Lahore, Pakistan; 4 Department of Medical Laboratories, College of Applied Medical Sciences, Qassim University, Buraydah, Saudi Arabia; 5 Department of Microbiology, Government College University, Faisalabad, Pakistan; University of Balochistan, PAKISTAN

## Abstract

Middle East respiratory syndrome (MERS-COV), first identified in Saudi Arabia, was caused by a novel strain of coronavirus. Outbreaks were recorded from different regions of the world, especially South Korea and the Middle East, and were correlated with a 35% mortality rate. MERS-COV is a single-stranded, positive RNA virus that reaches the host by binding to the receptor of dipeptidyl-peptides. Because of the unavailability of the vaccine available for the protection from MERS-COV infection, the rapid case detection, isolation, infection prevention has been recommended to combat MERS-COV infection. So, vaccines for the treatment of MERS-COV infection need to be developed urgently. A possible antiviral mechanism for preventing MERS-CoV infection has been considered to be MERS-CoV vaccines that elicit unique T-cell responses. In the present study, we incorporated both molecular docking and immunoinformatic approach to introduce a multiepitope vaccine (MEP) against MERS-CoV by selecting 15 conserved epitopes from seven viral proteins such as three structural proteins (envelope, membrane, and nucleoprotein) and four non-structural proteins (ORF1a, ORF8, ORF3, ORF4a). The epitopes, which were examined for non-homologous to host and antigenicity, were selected on the basis of conservation between T-cell, B-cell, and IFN-γ epitopes. The selected epitopes were then connected to the adjuvant (β-defensin) at the N-terminal through an AAY linker to increase the immunogenic potential. Structural modelling and physiochemical characteristic were applied to the vaccine construct developed. Afterwards the structure has been successfully docked with antigenic receptor, Toll-like receptor 3 (TLR-3) and *in-silico* cloning ensures that its expression efficiency is legitimate. Nonetheless the MEP presented needs tests to verify its safety and immunogenic profile.

## 1. Introduction

MERS-COV was found in the Saudi Arabia kindgom as a highly pathogenic beta coronavirus in 2012, causing extreme acute respiratory diseases [1–3]. However, present studies suggested that insectivorous bats (*Neoromicia capensis* and *Vespertilio superans)* are considered to be the source, dromedary camels and European hedgehog are natural host and source of human MERS-COV transmission [1, 4]. hDPP4 (Human dipeptidyl peptidase 4) was found to be MERS-COV receptor [5]. The MERS-COV (C Betacoronavirus) has the biggest genome of about 30-kb in size and 30,000 nucleotides (nt) in length, having positive-sense single-stranded RNA and 7 predicted ORFs (ORF3, 4a, 4b, 5, 8), two large replicase (ORFs) ORF1a & ORF1ab which encoded two proteases, 1^st^ is a papain-like protease (PL2pro) and the 2^nd^ is a 3C-like protease (3CLpro) [6]. The removal or decomposition of ORF3-5 proteins deregulates the response of the host and increases inflammation [7]. Four structural genes like a spike (S) and its receptor-binding domain responsible for host and virus interaction. Envelope (E) responsible for host cell recognition. Membrane (M), lower interferon (IFN) level in affected people, and nucleoprotein (N) responsible for RNA binding takes place during ribonucleocapsid formation by MERS-COV due to this protein [8]. According to phylogenetic analysis in 2016, it is suggested to have 182 full-length genomes [1, 9]. MERS-COV has infected about 2,100 people and has a fatality rate of about 35% [10, 11]. Most of the patients infected with MERS-COV had comorbidities like diabetes and considered to be more severe in patients with a weakened immune system [11]. MERS-COV syndrome symptoms, however, include cough, pneumonia, fever, shortness of breath, and diarrhea [3]. In the most severe condition, it also causes kidney infection [12]. As there is no therapeutic or vaccine available in market against MERS-COV.

With the advent of immunoinformatics, bioinformatics and molecular docking and simulation approaches, this century witnesses a remarkable advancement in the domain of vaccine development and design. The production rate of viral vaccines was significantly improved by approaches such as structural and reverse vaccinology [13]. Therapeutic approaches for Zika, MERS-CoV and Ebola virus were developed using *in-silico* peptide prediction in several studies [14–16]. Antigenicity of the desired protein can be accurately predicted using different online tools. To develop an effective subunit vaccine, various antigenic determinants are needed to be chosen. Adjuvants should also be attached to the final vaccine construct to trigger the immune-system and significantly enhance the immune responses [17]. Forecast of potential epitopes and the designing of Multi-Epitope Peptide (MEP) constructs that may provoke cell-mediated and humoral- immune responses becomes a new approach with the advances of bioinformatics [18, 19]. In comparison to conventional vaccines, MEPs have so many merits such as; they are affordable, harmless and efficient in rationally engineering the epitopes [20]. In this study, MEP was constructed to control MERS-COV. MEP consists of helper T lymphocyte (HTL) epitopes & cytotoxic T lymphocyte (CTL). Both CTL and HTL MEPs consider that the linear B-cell epitopes are overlapping [8]. Molecular modelling instruments for peptide-MHC complexes were then used to check their post-docking contact for the identification of potential applicants to develop the peptide vaccines. An immunoinformatic approach was applied to predict the B-cell and T-cell epitopes to develop subunit vaccines. For this purpose, recognition of continuous & non-continuous B-cells & CTL epitopes are necessary. The T cell epitopes are important to forecast, which can reduce the time, cost and requirements for experimental results [21].

## 2. Material and methods

**Fig 1:**
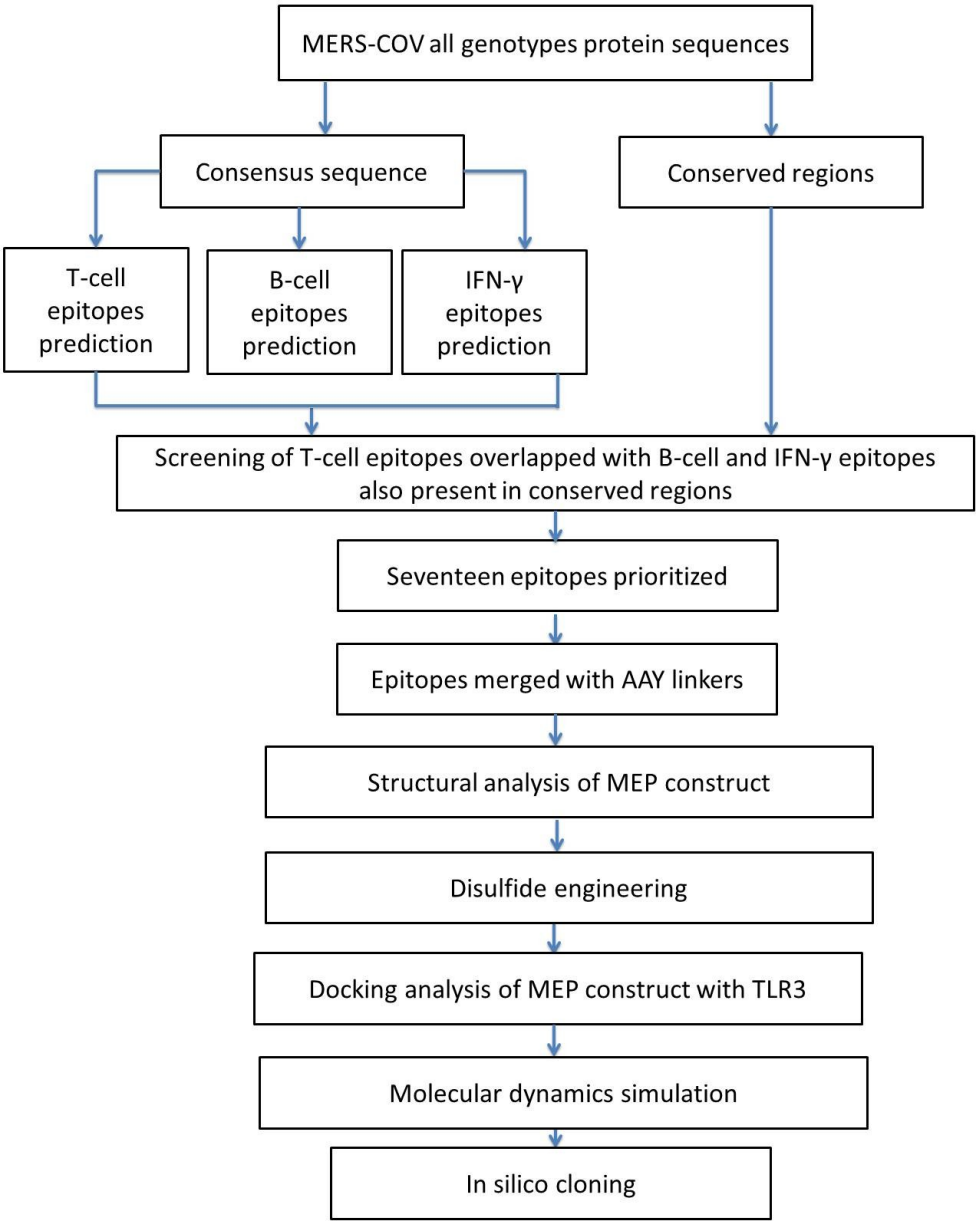
Overflow of complete work for vaccine construction.

The following four steps contribute to developing the present study: (1) T-cell epitopes (MHC class I & II) prediction against selected viral proteins from MERS-COV (ORF1a/1ab, NS3/3B/3C/3D/4A/4B/5, ORF3/4a/4b/5/8, membrane, nucleoprotein, spike, and envelope). (2) T-cell epitopes selection that is also present in conserved areas with expected B and IFN-γ epitopes. (3) Unification of selected epitopes using an appropriate linker & adjuvant to create MEP against MERS-COV with the help of structural modeling & epitope-epitope interactions depending on epitope combinations (4). The vaccine is characterized by TLR-3 molecular docking. The workflow of the work was shown in the **Fig 1**.

### 2.1. Data collection

Firstly, the proteome of MERS-COV was studied via literature [6]. Amino- acid sequence of structural (spike (AKL59401), membrane (AKL59407), Envelope (AKL59406) and nucleocapsid (AKL59408)) and non-structural proteins (ORF1a (A0A2P1ITC7) /1ab (A0A140AYZ4), NS3/3B/3C/3D/4A/4B/5, ORF3 (K9N796) /4a (K9N4V0) /4b (K9N643) /5 (K9N7D2) /8 (A0A0U2GQ91)) [8, 22] were obtained from Uniprot database in the FASTA format [23].

### 2.2. Sequence conservation analysis

Multiple sequence alignment (MSA) as well as positional diversity of sequences using CLC main workbench was carried out to perform sequence conservation assessment [24]. The resulting sequences were first aligned to achieve the consensus sequence and then the consensus-based sequences were aligned to achieve the conserved areas between all genotypes and an overall consensus sequence. Then variability of attained consensus sequences was checked via Protein Variability Server (PVS) [25]. All the retrieved consensus sequences were submitted to antigenic prediction. The antigenicity of sequences were checked via VaxiJen. Non-antigenic proteins were removed & antigenic peptides were then used for further analysis [26, 27].

### 2.3. Epitope screening

Several peptides (consensus) of all genotypes of MERS-COV the B-cell, T-cell (MHC class I & II) and IFN-γ stimulating epitopes were forecasted.

#### 2.3.1. Prediction of B-cell epitopes

B-lymphocytes are divided into memory cell and the plasma cells that secrete antibodies after contact with antigen (such as B-cell epitopes). Due to its hydrophilic nature, they are considered to more accessible for the flexible region [21]. The 20-mer linear B-cell epitopes of (M),(E), (N)) & (ORF1a, ORF3/4a/8) were predicted through the online IEDB immune epitope database [28]. In a linear B-cell sequence of proteins this computing programme generated genuine predictions.

#### 2.3.2. T-cell epitope prediction (MHC class I & II)

The nine-mer T-cell epitopes (MHC class-I & MHC class-II) were predicted through online server Propred1& Propred, respectively [29]. For prediction, all alleles were picked. Epitopes that will bind with a higher number of alleles are considered to be more immunogenic. In peculiar, bind with the alleles involved in MERS-COV protection were chosen. Both servers produce a critical result similarly to throughout computation. The prediction for class I allele proteasomes and immunoproteasomes continued and the threshold had been set at 5%.

#### 2.3.3. Interferon-γ inducing epitope prediction

IFN-γ may also be responsible for the immune response, both innate and adaptive, by producing T helper cells and cytotoxic T cells. It additionally up-regulates the epitopes of MERS-COV, (M),(E), (N)) and (ORF1a, ORF3/4a/8) against IFN-γ, predicted through IFN epitope server [30]. This server is available for users which could enable them to predict IFN-γ inducing peptides with capabilities of inducing IFN-γ.

### 2.4. Non-human homologues epitopes screening

To prevent autoimmunity, the vaccine candidate should not be like the human proteome. Thus, Blastp [31] was performed against the human proteome and identities >80% were then excluded.

### 2.5. Antigenicity of epitopes

Both structural and non-structural T cell ephitopes (MHC class I & II) for MERS-COV have been tested and overlapped with predicted IFN-γ-inducing and B-cell epitopes. Then the antigenicity was tested by means of an online tool Vaxijen at 0.5 threshold [32].

### 2.6. Prediction of physicochemical properties

ProtParam tool has been used to examine many physicochemical properties of MEP [33]. The Based on the pK of the amino acids concerned, the ProtParam method tests several physicochemical properties [34]. The half-life (in vivo) forecast is based on the N-end rule that determines protein degradation in N-terminal amino acids [35]. The stability of the proteins is evaluated by means of the Instability Index which shows the value < 40 reports for protein stability. The aliphatic index forecast the amount of the aliphatic side chains involved.

### 2.7. Secondary structure prediction

The PSIPRED server-has been applied to evaluate secondary structure of the MEP [36]. For the prediction, two feed-forward neural network methods were used. The 1st network forecast is used in order to provide the 2^nd^ network with the feedback to evaluate PSI-Blast performance and to refine the structure [37].

### 2.8. 3D structure prediction

The MEP’s 3D structure has been forecast using the I-TASSER web server [38]. The prediction was followed by refinement using a Galaxy Refine server which was freely accessible [39]. The method depends on the possibility of improving both the local and global structural quality. The refining needs to rebuild and repackage side chains, subjected to a molecular dynamic simulation involving recurrent structural disorder and relaxation. To avoid structural disruption, a three axis locking system was used [39]. The SAVES server was used to choose the best-developed model that was tested with WHATCHECK [40], ERRAT [41] and PROCHECK [42].

### 2.9. Vaccine stability through disulfide engineering

Before the next step, refined protein model’s stability should be improved. Disulfide bonds are covalent interactions emulating stable molecular interactions which, by conforming precision geometric conformations, ensure a significant stabilization of the protein model. Disulfide engineering is a modern way to form disulfide bonds in the target protein structure. Therefore, the refined MEP model was incorporated into disulfide by design 2.0 to achieve disulfide engineering [43]. Initially the refined protein model was tested for residue pairs which could be used in disulfide engineering. For mutation of cysteine residues using the mutated server feature, a pair of residues was picked.

### 2.10. Molecular docking of MEP & TLR3

Altogether, interaction among immune receptor molecule and the antigenic molecule is necessary for the effective evocation of immune response. A GRAMM-X on-line server that uses a refinement phase, smooth potential and knowledge-based scoring has been used to analyse the interaction among vaccine build & immune reception TLR-3 (PDB ID: 2A0Z). Pymol has been used for display and study of the model structure [44]. An online server PDBsum was used to achieve the standard sketches of interactions between docked-proteins. The protein-protein interactions among docked molecules were analyzed by it [45].

### 2.11. Molecular dynamics simulation (MDS)

The study of molecular dynamics is important when evaluating the stability of the protein-protein complex. Protein stability can be calculated by comparing the essential protein dynamics with normal mode. The iMODS server’s normal mode analysis (NMA) was used to define the collective protein movements in the internal co-ordinates. The server analyses the magnitude and direction of the immanent movements in regard to its eigenvalues, deformability, covariance, and B-factors. Deformation of main chain depends on the deformation of a certain molecule in each residue. The rigidity of the motion was defined by the eigenvalue of each normal mode. Eigenvalue is directly related to energy needed to deform the structure and the deformation is much easier if its value is low.

### 2.12. *In silico* cloning

The codon adaptation method was used to evaluate the reverse translation and codon optimization to confirm the translation efficiency of cloning and appropriate vector expression. In the form of graphical representation as well as codon adaptive index (CAI) values, it provides end products [46].

## 3. Results

### 3.1. Conservation profile of MERS-COV structural & non-structural protein

Conservation analysis is a common method for predicting residues that are functionally important in protein sequences [47]. The conserved regions amongst the selected nonstructural and structural proteins of all genotypes, conserved regions (described in Table 1) which is 4 in envelop protein, 4 in membrane protein, 6 for nucleoprotein, 10 and 4 regions for ORF1a and ORF8 respectively. Moreover, only 1 region was found to be conserved in ORF3 and ORF4a, respectively **(Table 1).** Applying this analysis, subsequent T-cell, IFN-γ, and B-cell epitopes were forecasted using these selected regions.

**Table 1:** Conserved regions in MERS-CoV (E), (M), (N), ORF1a/4a, ORF3/8.

Conserved regions (envelop protein)	Positions
MLPFVQ	1–6
LTATRLCVQC	34–43
YNTGRSVYVKFQ	59–70
SKPPLPP	72–78
Conserved regions (membrane protein)	Positions
MSNMTQL	1–7
YPIDLASQIISG	70–81
NEVTVAKPNVLIALKMVKRQS	165–185
GTNSGVAIYHRYKAGNYRSPPITADIELALLRA	187–219
Conserved Regions (nucleoprotein)	Positions
VGGDLLYLDLLNRLQALESGKVKQSQPKVITK	209–283
KDAAAAKNKMRHKRTSTKSFNMVQ	209–283
AFGLRGPGDLQGNFGDLQL	209–283
KLGTEDPRWPQIAEL	285–299
PTASAFMGMSQFKLTHQNNDD	301–342
HGNPVYFLRYSGAIKLDPKNP	301–342
Conserved Regions (ORF1a)	Positions
DKLRDYLADYDVAVTAGPFMD	520–540
AINVGGTGLQYAAITAPYVVLTGLGESFKKVATIPYKVC	542–580
SVKDTL	582–587
YYAHSVLYRVFPYDMDSGVSSFSELLFDCVDLSV	589–622
LDTCFEATEATFNFLLDLAGLFRIFLRNAYVYTSQGFV	655–725
VVNGKVSTLVKQVLDLLNKGMQLLHTKVSWAGS	655–725
ISAVIYSGRESLIFPSGTYYCVTTKAKSVQQDLDVILPGEFSKKQL	727–772
FVTTLTSDYTITVFVTVNLAKVCTYAIFAY	3722–3784
SPQLTLVFPEVKMILLLYTCLGFMCTCYFGVFS	3722–3784
LNLKLRAPMGVYDFKVSTQEFRFMTANNL	3786–3853
TAPRNSWEAMALNFKLIGIGGTPCIKVAAMQSKLTDLKC	3786–3853
Conserved regions (ORF3)	Positions
TGTQSVSVDRE	68–78
Conserved regions (ORF8)	Positions
KMLGIG	8–13
GMELSNWLPGG	23–33
DPKQHSHSGLLRMASFGSM	41–85
KMAPLMLLQLLGRGTLTMIQLLLHNS	41–85
Conserved regions (ORF4a)	Positions
MDYVSLLN	1–8

### 3.2. Epitopes prediction

T-cell epitopes for MHC class-I and II were forecasted by utilizing the conserved regions. The epitopes then screened based on the antigenicity and those were selected that binds to most of the alleles [48–50]. Furthermore, a prediction of 15-mer IFN-γ & 20-mer B-cell epitopes were carried out for each chosen area. Finally, the T-cell epitopes (MHC I & II)-overlapping with predicted IFN-γ and B-cell epitopes and present in conserved regions were then chosen. Moreover, Uniprot Blast investigates these epitopes, so that none are homologous to human proteins. Six epitopes displaying human protein homology were omitted. At the last, antigenicity of final epitopes was checked and those which show high antigenicity score were selected for further analysis **(Table 2).**

**Table 2:** Selected 15 T-cell (MHC I & II) epitopes among nucleoprotein, ORF8, and ORF1a proteins.

Epitopes (MHCI) (nucleoprotein)	Name	Antigenicity	Epitopes (MHCII) (nucleoprotein)	Name	Antigenicity
			MRHKRTSTK	N1	0.7535
			FMGMSQFKL	N2	0.9307
**Epitopes (MHCI) (ORF8)**			**Epitopes (MHCII) (ORF8)**		
MKMAPL	F1	2.1433	LLRMASFGSMKM	F2	0.8589
**Epitopes (MHCI) (ORF1a)**			**Epitopes (MHCII) (ORF1a)**		
GVFSLNLKL	R1	1.7616	VVLTGLGES	R3	0.6357
TCYFGVFSL	R2	0.6318	YVVLTGLGE	R4	0.7611
			LLFDCVDLS	R5	1.5000
			MALNFKLIGIGGTPCI	R6	1.7445
			MALNFKLIG	R7	1.8660
			VIYSGRESL	R8	0.8084
			YCVTTKAKS	R9	1.1122
			VTTKAKSVQ	R10	1.2284
			FKLIGIGGTPCI	R11	1.3412

**Fig 2:**
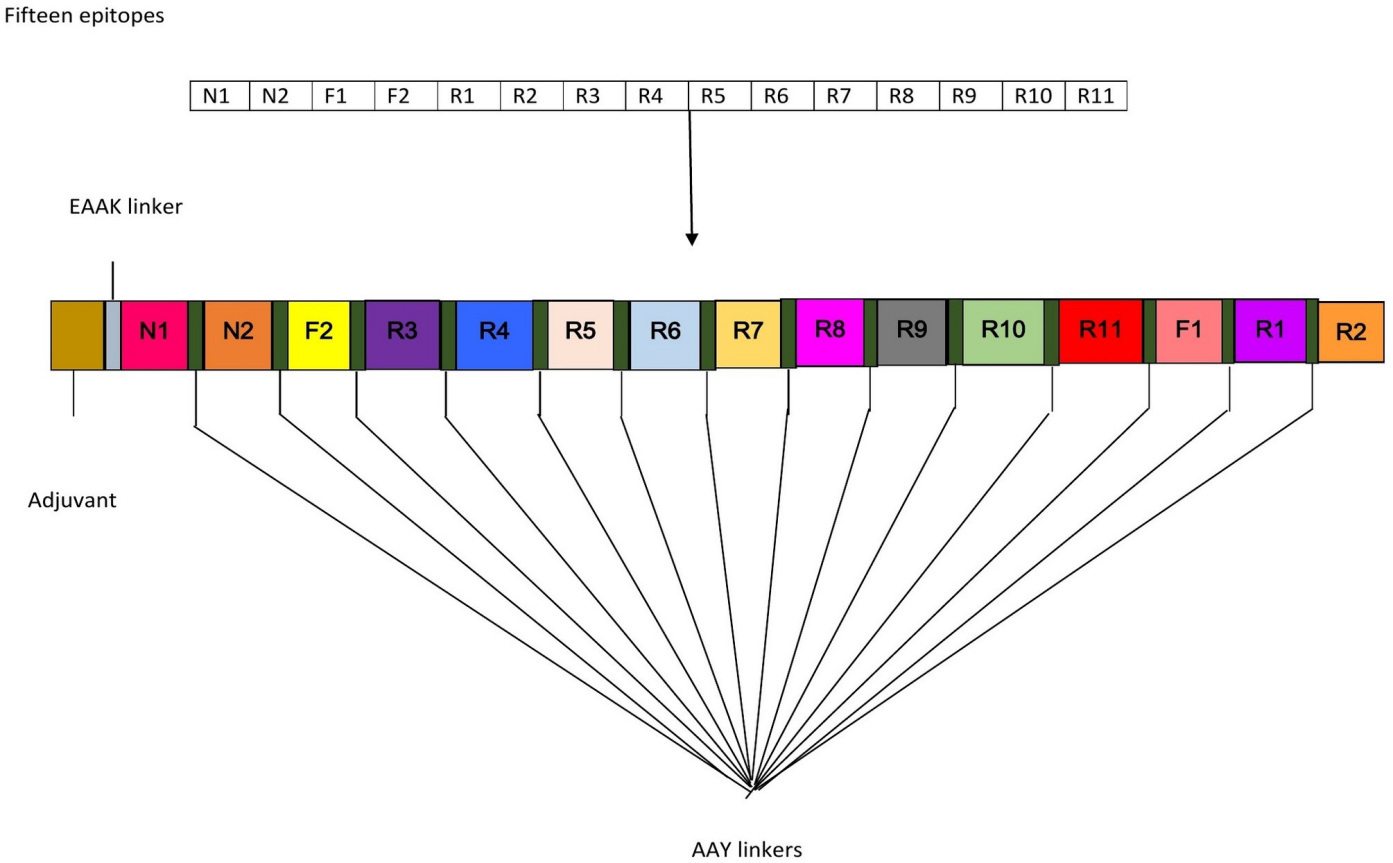
The vaccine construct’s schematic illustration consists of epitopes linked together by an adjuvant and linkers.

Eventually, the epitopes chosen for analysis hold the following properties: 1) T-cells epitopes presenting high score and potentially binds to numerous alleles. Furthermore, peptides have to be overlapping with B-cell and IFN-γ epitopes and must be non-human homologue and having high antigenicity as given in **Table 2**. Using the above-mentioned standards, only 15 epitopes were -prioritized for farther analysis. Among shortlisted epitopes, 2 were present in nucleoprotein, 2 in ORF8 and 11 in ORF1a **(Table 2).** None of the epitopes predicted for the envelope, membrane, and ORF3/4a fulfilled the criteria. For the designing of MEP, 15 epitopes (MHC class I & class II) were merged via flexible linker AAY. The peptide length discovered to be 187 amino acids. The AAY connector was employed to bind epitopes to effectively recognize the epitopes in the vaccine. MEP vaccines are poorly immunogenic and require pairing of adjuvants if used alone [51]. Adjuvants are vaccine formulation ingredients which protect against infection and influence immune responses, production, the durability and stability of the antigens [52]. Therefore, an adjuvant β-defensin (45 amino acids long) was integrated with the EAAAK linker at N-terminal. The EAAAK linker is used to insert the adjuvant and first epitope to permit an efficient separation of bi-functional fusion protein domains [53]. With the addition of adjuvants and linkers, the final stretch of the vaccine was found to be 237 amino acids, as seen in **(Fig 2).**

### 3.3. Physiochemical characteristics of the MEP vaccine construct

Various physicochemical characteristics of MEP constructs were calculated via the ProtParam server. The theoretical PI of construct was 9.68, describing it as basic, and its molecular weight is 25662.56 g/mol. The approximated aliphatic index is 89.54 describing it is thermostable. The estimated half-life of the construct was computed to be 30 hours *in vitro* (in mammalian reticulocytes), on the other hand, it is estimated to be >10 hours and >20 hours *in vivo* (*Escherichia coli* and yeast) respectively. The GRAVY value was measured at 0.381, showing its hydrophobic aspect with a positive value [54]. The instability index of the construct was computed to be 29.12 which indicate it as stable.

### 3.4. Secondary-structure prediction

For the secondary-structure prediction based on the amino-acids of the protein build, PSIPRED was used to predict secondary structure features (alpha-helix, β-strands & coils). Of the 237 amino acids, 113 are α-helix, which is 47.68%; 51 are β-strands of 21.52%; and 73 amino acids compose 30.80% of the overall MEP structure of the coils **(S1 Fig)**

### 3.5. Tertiary structure prediction and refinement

**Fig 3:**
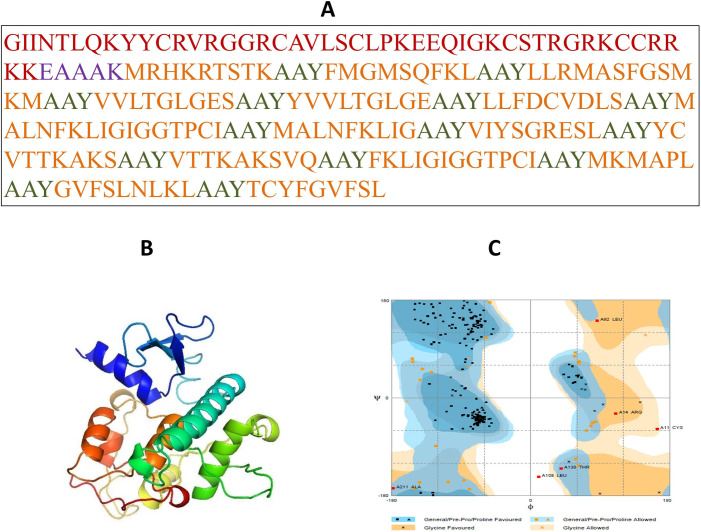
(A) A 237 amino acid long MEP sequence consisting an adjuvant (Maroon) linked at N-terminal with the help of EAAAK linker (Purple). MHC class I & II epitopes (orange) are joined by AAY linkers (Green). (B) The predicted three- dimensional structure of the MEP vaccine construct. (C) Ramachandran plot analysis of MEP structure where 86.0% 11 of residues present in the most favourable region.

Using the I-TASSER server, the tertiary structure of the MEP-construct was predicted. The amino acid sequence was provided as the input for the top 10 threading templates used by I-TASSER. The top five models were created through I-TASSER respectively from which the third model (model 3) was selected, which demonstrates C-score value about -4.47 depicting the high quality of the selected model. Furthermore, the model must be indicated 3661 atoms, 3695 bonds, 237 groups and one chain present in it respectively. Moreover, I-TASSER predicts the ligand-binding site in the structure. The predicted model was displayed in (**Fig 3B**). Besides, to raise the quality of the anticipated model, it was subjected to the GalaxyRefine server for refinement. The refined model exhibited 86.8% favored region in Ramachandran plot and displayed RMSD-as 0.498, MolProbity as 2.627, clash score-as 21.8 & poor rotamers-as 1.7. Thus model 3 was picked out for further analysis as shown in. SAVES server was manipulated for the selection of the best-established structure. SAVES server assessed the designed models by envisioning through ERRAT [41], WHATCHECK [40] and PROCHECK [42] results of SAVES analyses. SAVES server distinguished that the model 4 designed by GalaxyRefine was the best one. According to the Ramachandran plot, 86.0% of residues lie in the most favored region, 11.5% of amino acids reside in the generously allowed region while 2.6% are exhibited in disallowed regions (**Fig 3C**).

### 3.6. Disulfide engineering for vaccine stability

**Fig 4:**
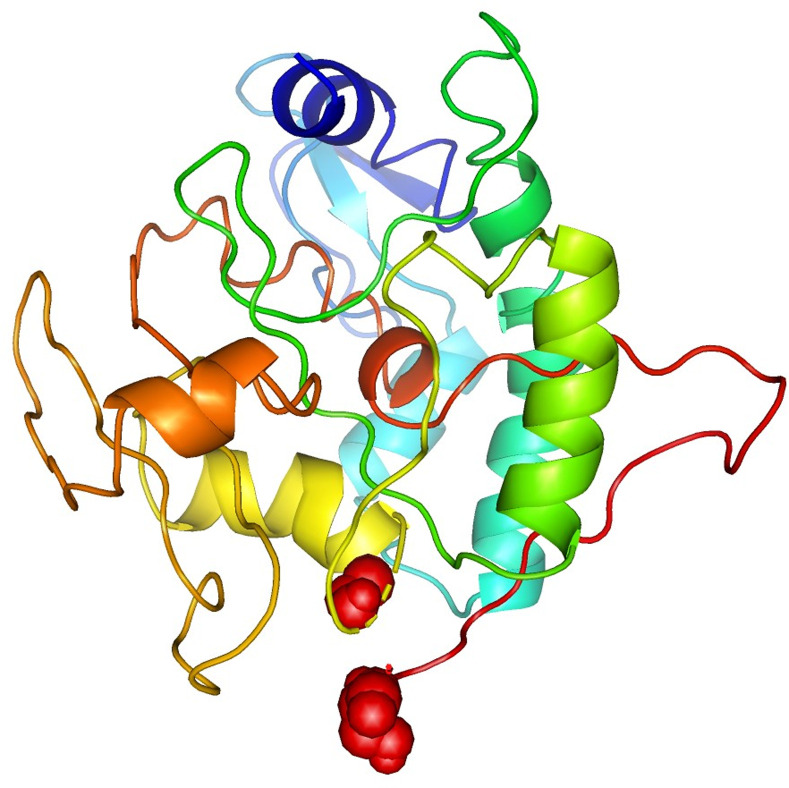
Disulfide engineering to improve protein stability. Displaying 1 magenta coloured mutated residue pair. These residues were selected based on their chi3 value, B-factor, and energy.

Disulfide by designv2.036 was used to perform disulfide engineering to stabilize the model structure of MEP construct. For disulfide engineering purposes, a total of 45 residue pairs can be used. Only 1 residue pair was finalized after parameters evaluation such as Chi3 value and energy because its value fall below the allowed range i-e Chi3 should be between -87 and +93 degrees and the energy value should be below 2.2. A total of 2 mutations were therefore created in the residue pair i-e Gly136-Phe235 (**Fig 4**).

### 3.7. MEP-TLR3 docking

**Fig 5:**
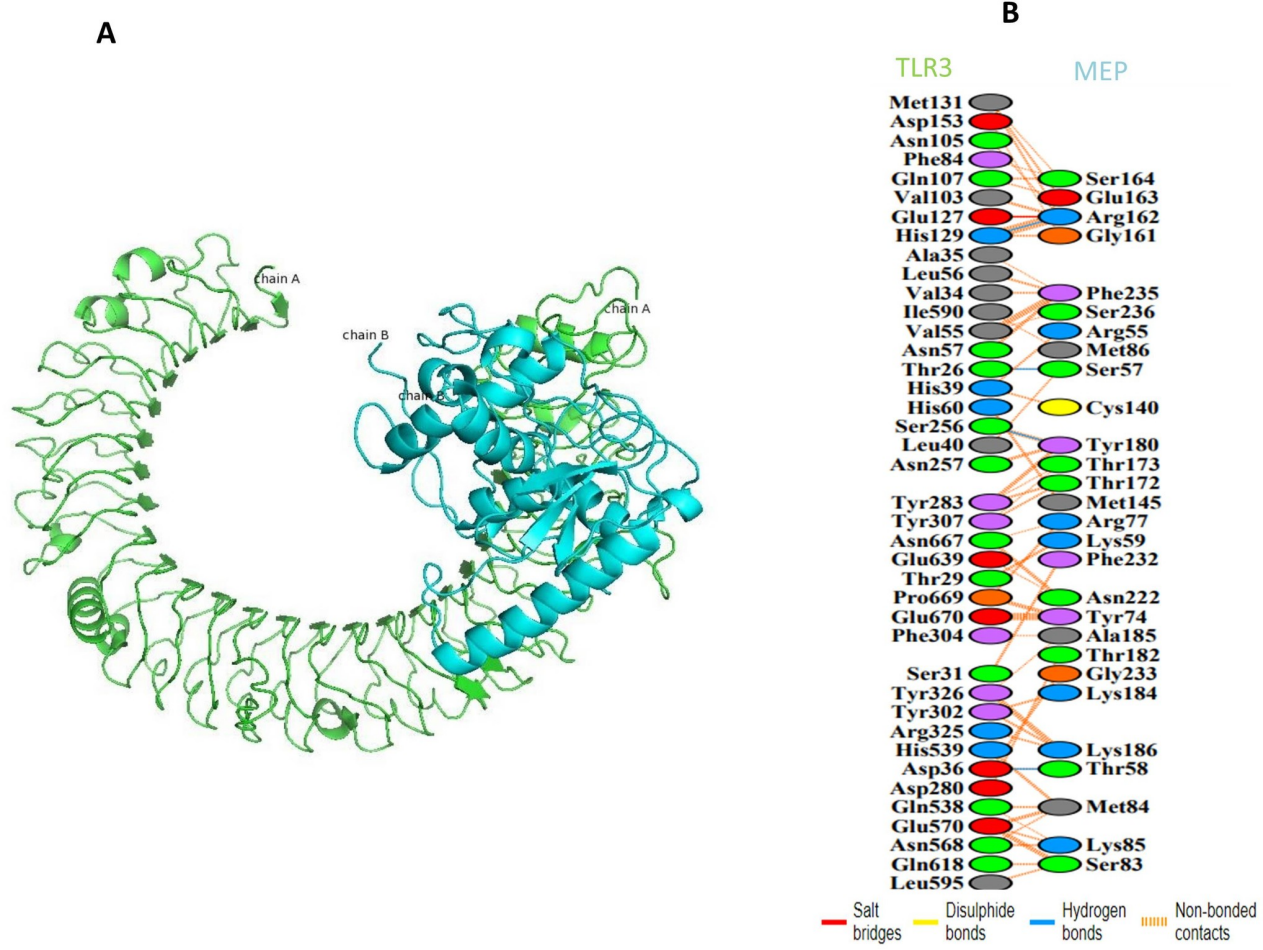
MEP construct docking with human TLR3: (A) MEP-TLR3 docked complex in cartoon representation. MEP vaccine construct is represented with blue colour and TLR3 is shown in green colour. (B) All interacting residues of TLR3 and MEP. 4 Hydrogen bonds with blue lines are indicated. TThe colours of the residues interacting reflect amino acid properties (Negative: Red, Positive: Blue, Neutral: Green, Aromatic: Pink, Pro&Gly: Orange, Cys: Yellow, and Aliphatic: Grey).

A suitable interaction among the antigenic molecule and the immune receptor molecule is required to start the immune response. Hence, the GRAMMX server was used for the success of the MEP-construct docking-analysis with the TLR3. TLR3 is showed in green colour in the docked complex, while the MEP structure is shown in blue colour, as shown in (**Fig 5A**). PDBsum was used to achieve the standard sketches of interactions between docked-proteins. It brought forth a schematic characterization of hydrogen-bond & nonbonded interactions among the docked proteins-complex. Our MEP construct had 4 hydrogen bond-interactions [Chain A-(TLR3)-B-(MEP construct); 129–162, 26–57, 256–180, and 36–58] with TLR3. The structural analysis brought out that HIS 129 and ARG 162 at a distance of 3.00 Å formed hydrogen bond, THR 26 and SER 57 formed hydrogen-bond at a distance of 2.89 Å, SER 256 and TYR 180 at a distance of 3.07 Å formed hydrogen bond, while ASP 36 and THR 58 formed hydrogen bond at a distance of 2.31 Å (**Fig 5B**).

### 3.8. Molecular dynamics simulation (MDS)

**Fig 6:**
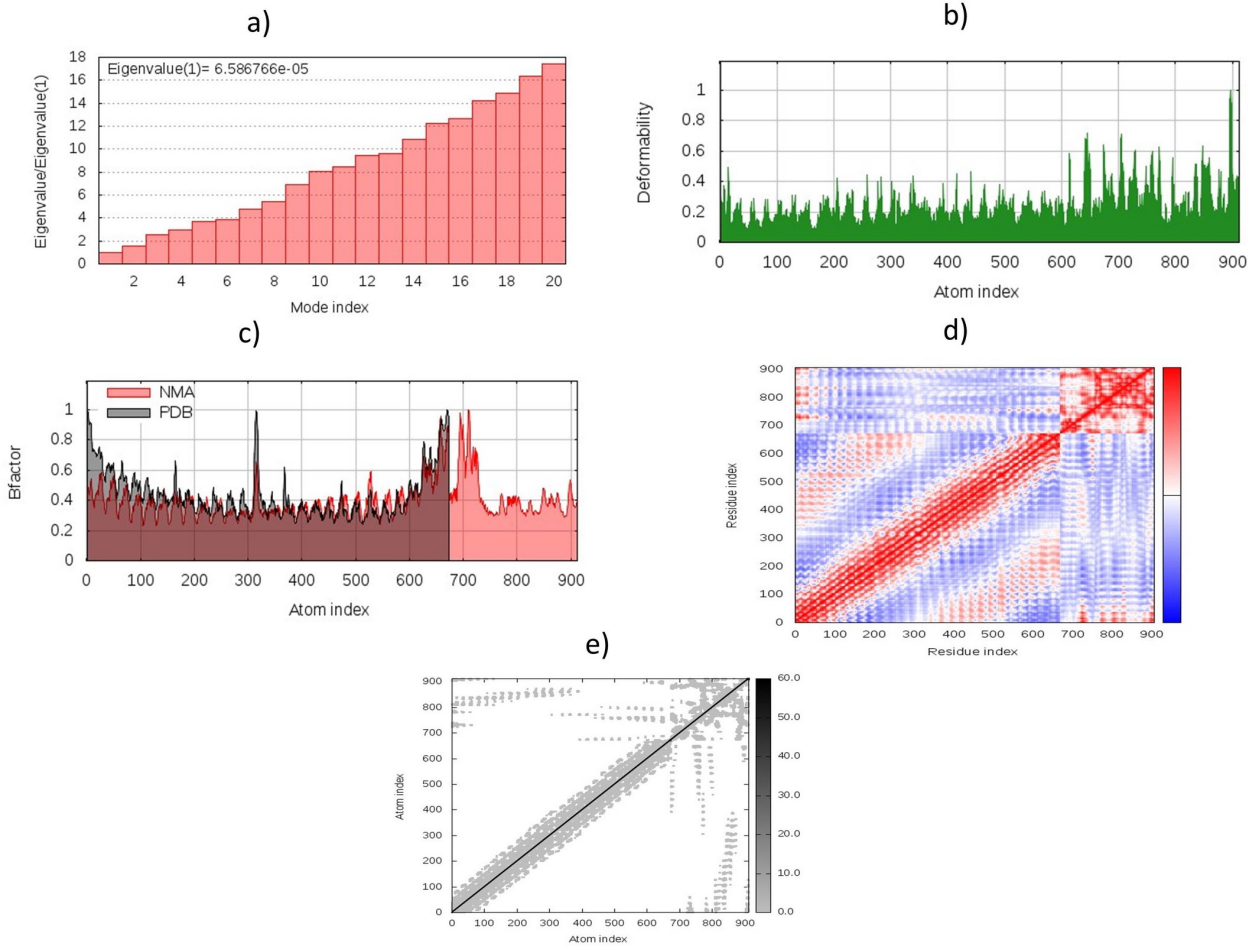
Molecular dynamics simulation of the MEP-TLR3 complex, showing (a) eigenvalue; (b) deformability; (c) B-factor; (d) covariance matrix; and (e) elastic network analysis.

Normal mode analysis (NMA) was conducted to scrutinise the mobility and stability of proteins on a wide scale. The iMODS server conducted this evaluation based on the internal coordinates of the docked complex. The deformation of the complex depends on the each residue’s distortion, as illustrated by chain hinges **(Fig 6B)**. The complex’s eigenvalue was found to be 6.586766e−05 **(Fig 6A)**. The variance with each normal mode was reversed to the eigenvalue. B-factor values were proportional to RMS, produced by normal mode analysis **(Fig 6C)**. The combination of residual pairs was defined by the covariance matrix showing several pairs of uncorrelated, anti-correlated, and related movements represented by white, blue and red colours **(Fig 6D)**. It also resulted in an elastic network model that distinguished the atom pairs linked by springs **(Fig 6E)**. Each point in the diagram shows the colour of the corresponding pairs of atoms by the grade of rigidity. The darker the colour, the stiffer the springs were.

### 3.9. *In-silico* cloning

The codon adaptation method was used to generate a cDNA sequence followed by an analysis of codon optimization based on GC content and adaptive index of codons (CAI). The MEP’s GC content was estimated as 66.53% that occupies the ideal range (30–70%), CAI was predicted as 0.96 that also exists within the ideal range (0.8–1.0) that describes the high protein expression that enhances its reliability.

## 4. Discussion

The highly effective prophylactic approach to increasing public health standards and preventing the spread of infection is vaccination. The computation tools were utilized for peptide-based epitopes prediction representing one of the important stages of designing vaccines [55]. The recent progress has been aimed at enhancing traditional controls to test T-cell responses against multiple vaccine candidates [56].

Immunoinformatics techniques play a critical role in the design of B-cell & T-cell epitope prediction subunit vaccines [57]. Researchers used immunoinformatics to provide innovative multi-epitope driven vaccine models for the ZIKV, SARS-COV-2, Ebola virus etc [58–60]. Detection of helper T-cell (HTL), CTL and B-cell epitopes are essential steps for MEP development [61]. The development of the MEP construct against MERS-CoV yielded hope full results while retaining the therapeutic authority and defensive approach of the designed vaccines candidate [62]. So far, the innovative -T-cell epitopes-based vaccinology strategy has produced positive results against diseases such as MERS syndrome and cancer and has demonstrated the strong immunogenicity in triggering T-cell responses [63]. The current objective is the development of a multiepitope T-cell vaccine against MERS-CoV syndrome. In this study, the multiple-epitope vaccine was designed that might be given protective immunity against MERS-CoV. MEP’s are beneficial as compared to the monovalent vaccines as they can evoke both humoral and cellular immunities [64]. During the severe course of MERS-CoV infection, unique T-cell responses are evoked towards non-structural MER-CoV proteins, so the response tends towards structural MERS-CoV proteins during infection [65]. Therefore both structural and non-structural MERS-CoV proteins for MEP vaccine construction are included in this report. Briefly, conserved T-cell epitopes were predicted by multiple sequence analysis followed by prediction of IFN-γ & B-cell epitopes. T-cells were tested to be overlapping with IFN-β and B-cell epitopes after the prediction was completed. The antigenicity of these epitopes were then evaluated. Both T-cell & B-cell reactions are triggered by the chosen overlapped regions. The vaccine construct can also increase the IFN-α reaction, inducing T helper cells to be very effective at elevating strong immune reactions [66]. It is proposed that conserved epitopes and host-specific non-homological ones may avoid barriers to epitope-based vaccines [67]. Few of the epitopes predicted in our study are the part of determined regions, predicted in the study of MEP construction against MERS performed by *Srivastava*, *S*., *et al*, such as GDLLYLDLLNRLQAL and MDYVSLLN in nucleoprotein and ORF4a respectively [68]. The combination of adjuvant in build MEP vaccines was intended by multiple adaptive & innate immune intermediaries to enhance immunogenicity activity [69].

The study of the MEP construct’s physiochemical characteristics has shown that it is basic, hydrophobic and stable. According to the theoretical pI, MEP was found to be basic value so it ensures a stable physiological pH interaction. The determined aliphatic index and scores for the instability index suggested that the protein of the vaccine could be stable and thermostable. The positive GRAVY score indicates its hydrophobic nature. It was found that MEP was immunogenic, non-allergenic, and highly antigenic. This suggests the epitopic vaccine’s ability to produce a robust immune response with no allergic reactions.

The 3D structure prediction offers a detailed understanding of the spatial arrangement of essential protein components. It provides excellent support for the study of protein functions, other protein components, interactions with ligands and dynamics. After refinement, the desirable features of the MEP structure have improved considerably. The plot analysis of Ramachandran demonstrates that the majority of residues are present in favoured and permitted areas, with very few residues in the disregarded sector, showing the model to be of good overall quality. The good quality of the engineered MEP construct is further indicated by Poor Rotamers, RMSD value, MolProbity and Clash Score. During this work we introduced disulfide bridging in the MEP structure to modify its functional properties, aid in the study of genetic components and increase protein thermostability.

The final structure thus formed was then docked against TLR3 to test adequate binding to immediate immune response. It has been proposed that TLR3 enhances the infection’s antiviral mechanism and helps track dead infectious cells [70]. To effectively transport the vaccine into the body, the strong binding affinity of MEP-TLR3 is needed.

MDS has been conducted to determine the stability and potential immune interactions between TLR3 and MEPs. Energy minimization has been performed to minimise the energy capacity of the docked MEP/TLR 3 system-wide complex to achieve maximum structural stability. Energy minimises inadecuate structural geometry, eliminates the proper stereochemistry and stabilises the structure of individual protein atoms. The derived eigenvalue shows the rigidity of the movement and energy necessary for the complex deformations.

Due to the incompatibility of mRNA codons requiring optimum codon expression, the effectiveness of the host translation of foreign genes varies [71]. The resulting CAI value was 0.96 and the GC content was also within an acceptable limit of 66.53%, implying possible higher expression in the *E*.*coli* K-12 system.

The study aimed to create a landmark in the production of MEP vaccines against MERS-CoV infection. To build a MERS-CoV MEP construct, which is a new scheme so far the research incorporating non-structural and structural proteins of MERS-CoV. Moreover, efficient docking of antigenic receptors with the MEP construct has significantly improved this study’s accuracy and scope. A carefully built MEP can therefore become an important asset in the battle against viral contamination and tumours. Recent research indicates that the engineered vaccine could be tested experimentally (in vitro and in vivo) to develop a possible MERS-CoV infection vaccine.

## 5. Conclusion

This study employed a variety of approaches, such as immunoinformatics, for the building of MEPs that could be protective and carry an immunogenic potential that could therefore elicit both types of responses. This study justifies further experimental validation despite the computational prediction and the confirmation of epitopes.

## Supporting information

S1 Fig Schematic illustration of secondary structure prediction of vaccine construct through PSIPRED. (TIF)
