# Supplementary figures and images for: Rational design of multi epitope-based subunit vaccine by exploring MERS-COV proteome: Reverse vaccinology and molecular docking approach
Source: PLoS One. 2021 Feb 3;16(2):e0245072. doi: 10.1371/journal.pone.0245072 (PMC7857617; doi:10.1371/journal.pone.0245072)

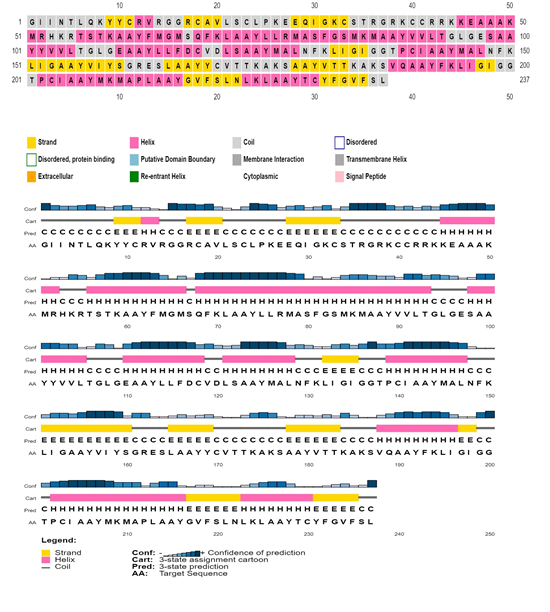

Supplement: S1 Fig — (TIF) [file pone.0245072.s001.tif]
